# Reassessment of extracellular and intracellular activity of macrolides, rifampicin, and doxycycline against *Rhodococcus equi* based on bacterial counts and microscopy

**DOI:** 10.1128/spectrum.01205-25

**Published:** 2025-07-29

**Authors:** Anne-Sophie Huguet, Ophélie Gourbeyre, Alain Bousquet-Mélou, Aude A. Ferran, Elodie A. Lallemand

**Affiliations:** 1 INTHERES, Université de Toulouse, INRAE, ENVT 27091, Toulouse, France; Michigan State University, East Lansing, Michigan, USA

**Keywords:** macrophages, foal, drug combination, lung cells

## Abstract

*Rhodococcus equi* is an opportunistic intracellular pathogen responsible for severe respiratory infections in foals, requiring prolonged antibiotic treatment. Despite its *in vitro* susceptibility to multiple antibiotics, the *in vivo* eradication of intracellular *R. equi* remains challenging due to its ability to persist and survive within alveolar macrophages. This study aimed to assess *in vitro* the extracellular and intracellular activity of rifampicin, doxycycline, and four macrolides against *R. equi* while assessing current methodologies used to differentiate intracellular and extracellular bacteria. We found that the removal of extracellular *R. equi* with short times of exposure to bactericidal drugs was challenging. We thus proposed an alternative approach with washing steps to assess intracellular antibiotic activity by combining bacterial counts and optical microscopy. Our results indicated that clarithromycin alone, as well as azithromycin or gamithromycin in combination with doxycycline, dramatically reduced both intracellular and extracellular *R. equi*. This study also highlighted the need for refined *in vitro* methodologies to assess antibiotic efficacy against *R. equi,* ultimately contributing to the development of optimized treatment strategies for *R. equi* infections in foals.

**IMPORTANCE:**

*Rhodococcus equi* is a major cause of pneumonia in foals and represents a serious challenge for the equine sector due to the need for prolonged and sometimes ineffective antibiotic treatments. This study highlights the limitations of current *in vitro* methods used to evaluate antibiotic activity against *R. equi*, by demonstrating that widely used bactericidal antibiotics like gentamicin may not eliminate extracellular bacteria as effectively as previously assumed, and we emphasize the risk of overestimating intracellular bacterial survival in experimental models. We therefore propose a new method that allows more accurate assessment of antibiotic efficacy within host cells. Our findings help refine *in vitro* testing strategies and support the identification of antibiotic combinations with effective intracellular activity, offering new perspectives for improving treatment protocols against *R. equi* infections in foals.

## INTRODUCTION

*Rhodococcus equi* is a bacterium commonly found in the environment and the gastrointestinal tract of horses. This opportunistic pathogen causes a severe respiratory disease that primarily affects young foals (1–3). The disease manifests as chronic pneumonia, pulmonary abscesses, and potential extrapulmonary complications, such as vertebral osteomyelitis or ocular infections (4). Its significant economic burden on the equine industry stems from the prolonged antibiotic treatment that is required, often involving rifampicin combined with a macrolide, and the absence of a reliable vaccine.

A crucial virulence factor of *R. equi* is its capacity to survive and replicate within host alveolar macrophages by forming *R. equi*-containing vacuoles. This intracellular survival strategy is largely facilitated by the plasmid-encoded virulence-associated protein A (VapA), a surface protein (5–7). Within macrophages, *R. equi* can survive in various microenvironments, including the cytosol, phagosomes, and phagolysosomes, each characterized by distinct pH levels and drug concentrations that can affect antibiotic activity (8). Indeed, *in vitro* studies have demonstrated that *R. equi* is susceptible to multiple antibiotics outside cells (9, 10), while their clinical effectiveness is often hindered by limited activity within cells. To predict intracellular antibiotic activity, we conducted a previous study by exposing *R. equi* to an acidic environment and antibiotic concentrations achievable in cells (11). However, the pH of *R. equi*–containing vacuoles remains uncertain (12, 13) due to the potential exclusion of the proton-pumping vacuolar ATPase complex from the phagosome by the virulence factors. As a result, directly evaluating, on phagocytized bacteria, the efficacy of antibiotics classically used against *R. equi* infections, namely rifampicin, doxycycline, clarithromycin, azithromycin, gamithromycin, and tulathromycin, is crucial for developing effective treatment strategies. To date, only one study has assessed the efficacy of rifampicin, doxycycline, azithromycin, and clarithromycin against intracellular *R. equi* (14). However, that study tested concentrations achievable in plasma, which were lower than those that can be reached in the pulmonary epithelial lining fluid (PELF). Moreover, studying the activity of an antibiotic separately in extracellular and intracellular environments presents a challenge, as it requires distinguishing between intracellular and extracellular *R. equi*. To remove extracellular bacteria, some studies investigating antibiotic efficacy, immune response, or virulence mechanism have used bactericidal agents, such as gentamicin, vancomycin, and amikacin (13, 15–18). However, the ability of these antibiotics to remove extracellular bacteria under the brief exposure conditions typically reported (1 h) has not yet been demonstrated on *R. equi,* which has a far lower replication time than *E. coli* or *S. aureus* (19, 20).

To assess *in vitro* the extracellular and intracellular activity of antibiotics, classically used against *R. equi* infections at concentrations achievable in PELF in foals, the study first evaluated these established protocols, including the use of gentamicin, vancomycin, and amikacin, to remove extracellular bacteria. Then, we proposed a preliminary alternative approach, including washing steps to discriminate between intracellular and extracellular activity of rifampicin, doxycycline, clarithromycin, azithromycin, gamithromycin, and tulathromycin on *R. equi*.

## MATERIALS AND METHODS

### Bacterial strain, cell culture medium, and MIC determination

The virulent strain *R. equi* ATCC 33701 was used in this study. The presence of the *vapA* gene was verified through PCR amplification as described previously (5). THP-1 cells (ATCC TIB-202) were cultured in RPMI supplemented with 10% heat-inactivated fetal bovine serum (FBS) and maintained in 75 cm² plastic tissue culture flasks at 37°C and 5% CO_2_.

Clarithromycin, azithromycin, gamithromycin, tulathromycin, and rifampicin were obtained from Glentham (Corsham, UK), while doxycycline, vancomycin, gentamicin, and amikacin were sourced from Sigma (Saint-Quentin-Fallavier, France). Stock solutions were prepared in water for doxycycline, vancomycin, gentamicin, and amikacin, and in methanol for rifampicin, clarithromycin, azithromycin, gamithromycin, and tulathromycin.

The MIC of gentamicin, vancomycin, and amikacin was determined using the standardized microdilution method recommended by the Clinical Laboratory Standards Institute, except for the broth, which was RPMI supplemented with 10% FBS (21). Briefly, a bacterial inoculum of 5 × 10^5^ colony-forming unit (CFU)/mL was prepared and exposed to serial twofold dilutions of antibiotics in 96-well plates. The MIC was assessed after incubation for 24 h at 37°C and 5% CO_2_. All MIC determinations were performed in triplicate.

### Removal of extracellular bacteria with bactericidal drugs (gentamicin, vancomycin, and amikacin): time-kill studies

Time-kill studies were performed in RPMI supplemented with 10% FBS to assess the bactericidal activity of gentamicin, vancomycin, and amikacin against extracellular *R. equi*. The tested antibiotic concentrations corresponded to those used in previous publications to eliminate extracellular *R. equi* (13, 15–18): 10 and 100 µg/mL for gentamicin, 3 µg/mL for vancomycin, and 8 µg/mL for amikacin. Briefly, for each TKS, a bacterial inoculum of 2 × 10^5^ CFU/mL was prepared from an overnight culture. Bacteria were exposed to the respective antibiotics and incubated at 37°C with 5% CO_2_ for 24 h. At predetermined time points (0, 1, 2, 3, 4, 5, 6, 7, 8, and 24 h), 100 µL of the bacterial suspension was collected, centrifuged, and washed with sodium chloride. The samples were then subjected to 10-fold dilutions, which were plated on Tryptic Soy Agar (TSA) supplemented with charcoal and magnesium sulfate. The plates were incubated overnight at 37°C; then, bacteria were counted. The limit of quantification (LOQ) was 1.8 log_10_ CFU/mL. All TKS experiments were conducted in triplicate.

### Macrophage infection

THP-1 cells were suspended at a density of 5 × 10^5^ cells/mL in a 25 cm² flask containing 10 mL of suspension and infected with 5 × 10^5^ *R. equi*/mL, prepared from an overnight culture in RPMI + 10% FBS. To facilitate phagocytosis, the cells and bacteria were co-incubated for 6 h at 37°C with 5% CO_2_ before the addition of antibiotics at the concentrations equivalent to the maximum achievable in PELF of foals and reported in Table 1. Rifampicin, doxycycline, and four macrolides—clarithromycin, azithromycin, gamithromycin, and tulathromycin—were first tested individually, and then in pairwise combinations: rifampicin with each macrolide, and doxycycline with each macrolide. Then, cells were incubated for an additional 40 h. A negative control with THP-1 cells was maintained without *R. equi* or antibiotics for 46 h, while a positive control included cells infected with *R. equi* and without antibiotics.

**TABLE 1:** The tested antibiotic concentrations (in µg/mL) based on the maximum concentration attained in the pulmonary epithelial lining fluid of foals reported in the literature

	Tested concentrations (µg/mL)
Rifampicin (22)	10
Doxycycline (23)	10
Azithromycin (24)	10
Clarithromycin (25)	50
Gamithromycin (26)	5
Tulathromycin (27)	2

### Assessment of activity of antibiotics against *R. equi* after macrophage infection

After incubation for 40 h with antibiotics, 100 µL of the suspension was collected in flasks to first count the total bacteria (i.e., extracellular and intracellular bacteria). The collected cells were centrifuged and subsequently resuspended in 1 mL of sterile water to lyse the THP-1 cells and to release the intracellular bacteria. Total *R. equi* was counted as described above for the TKS. The limit of quantification was 1.8 log_10_ CFU/mL.

In a second step, to better assess the activity of antibiotics against intracellular bacteria, the cells in the remaining suspension in the flask were washed three times in phosphate-buffered saline (PBS) with centrifugation at 1,300 rpm at 4°C to remove as many extracellular bacteria as possible. Fifty microliters of this suspension was cytocentrifuged onto glass slides and stained with May-Grünwald-Giemsa (MGG) stain to visualize bacteria within cells using microscopy. One milliliter was used to confirm the bacterial counts after washing. To do this, the suspension was centrifuged once again, resuspended in 1 mL of sterile water, and processed for bacterial counting, as described previously. The limit of quantification for intracellular bacteria was 1.1 log_10_ CFU/mL. All infection experiments were performed in triplicate.

### Statistical analysis

Statistical analysis was conducted using GraphPad Prism (version 8.4.2, GraphPad Software, San Diego, CA, USA). Analysis of variance was used to compare the effects of the antibiotics, with statistical significance set at *P* < 0.05.

## RESULTS

### MIC determination

The MIC of gentamicin, vancomycin, and amikacin for the *R. equi* ATCC 33701, determined in RPMI + 10% FBS, was 0.5, 0.25, and 1 µg/mL, respectively.

### Assessment of the ability of bactericidal drugs (gentamicin, vancomycin, and amikacin) to remove extracellular *R. equi*

**Fig 1:**
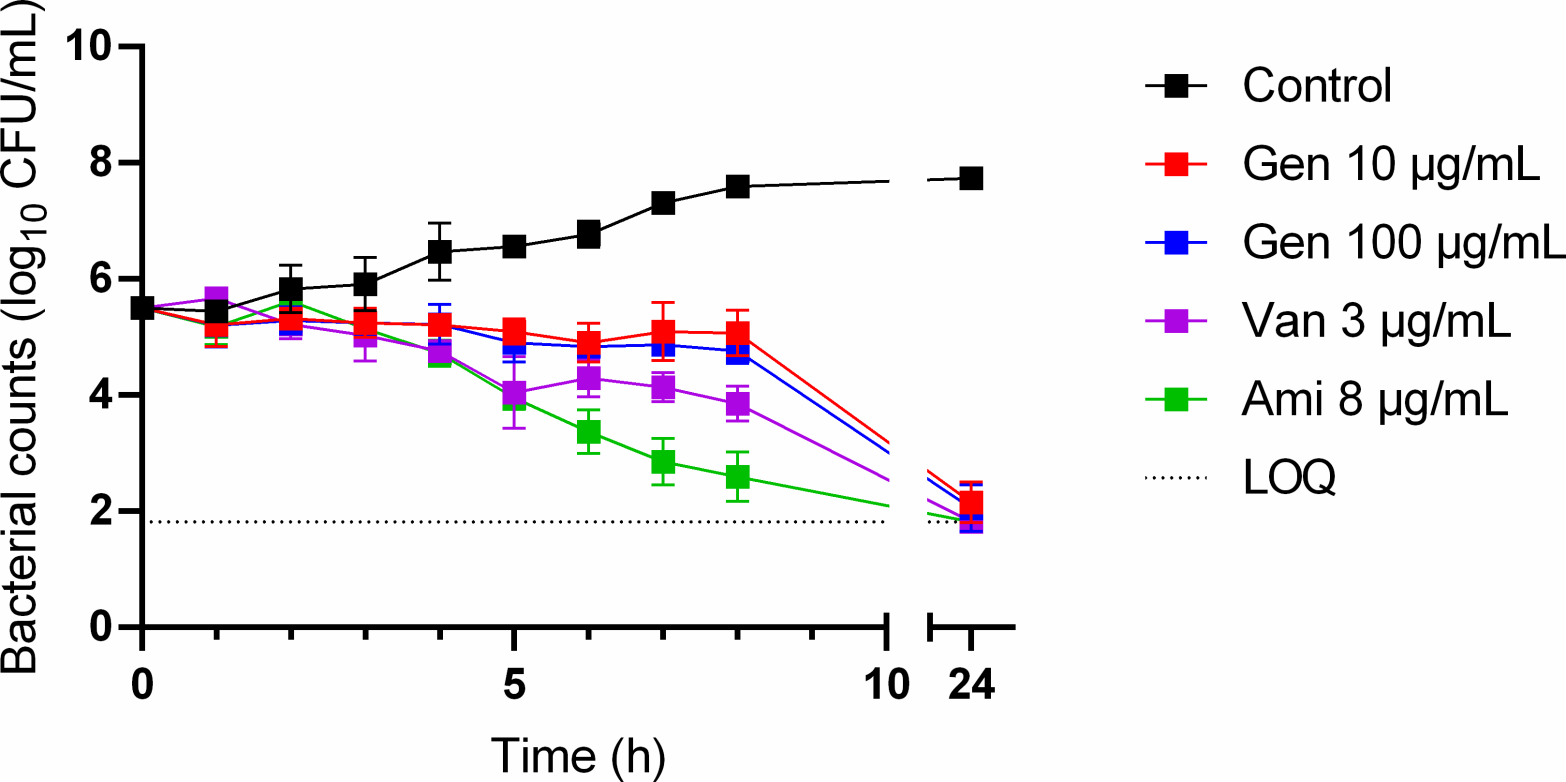
Counts of *Rhodococcus equi* ATCC 33701 in RPMI supplemented with 10% FBS following exposure to gentamicin (Gen, 10 and 100 µg/mL), vancomycin (Van, 3 µg/mL) or amikacin (Ami, 8 µg/mL). Each point on the curve represents the mean value of three independent experiments. LOQ, limit of quantification.

The antimicrobial activity of gentamicin at 10 and 100 µg/mL, vancomycin at 3 µg/mL, and amikacin at 8 µg/mL against *R. equi* ATCC 33701 was assessed through time-kill studies over a 24-h period to check whether extracellular bacteria could be removed reliably by these antibiotics. The results, obtained in RPMI + 10% FBS, are presented in Fig. 1. Within the first hour of exposure, none of the antibiotics had reduced the bacterial population. After 5 h, both vancomycin and amikacin had reduced the bacterial count by approximately 1 log_10_ CFU/mL. After 8 h, amikacin had achieved a 2 log_10_ CFU/mL reduction, while vancomycin had produced a reduction of around 1.5 log_10_ CFU/mL. Gentamicin demonstrated bacteriostatic activity at both tested concentrations (10 and 100 µg/mL) throughout the 8-h period. At 24-h post-infection, vancomycin and amikacin had effectively eradicated the bacteria, reducing the bacterial load to below the limit of detection. In contrast, gentamicin had not completely eradicated the bacteria, with a count remaining just above the lower limit of quantification.

Due to the lack of efficacy of these antibiotics against extracellular *R. equi* within 1 h of exposure, we sought a new approach to assess separately the activity of drugs on intracellular and extracellular bacteria.

### Assessment of the centrifugation and washing protocol efficiency to remove extracellular *R. equi*

**Fig 2:**
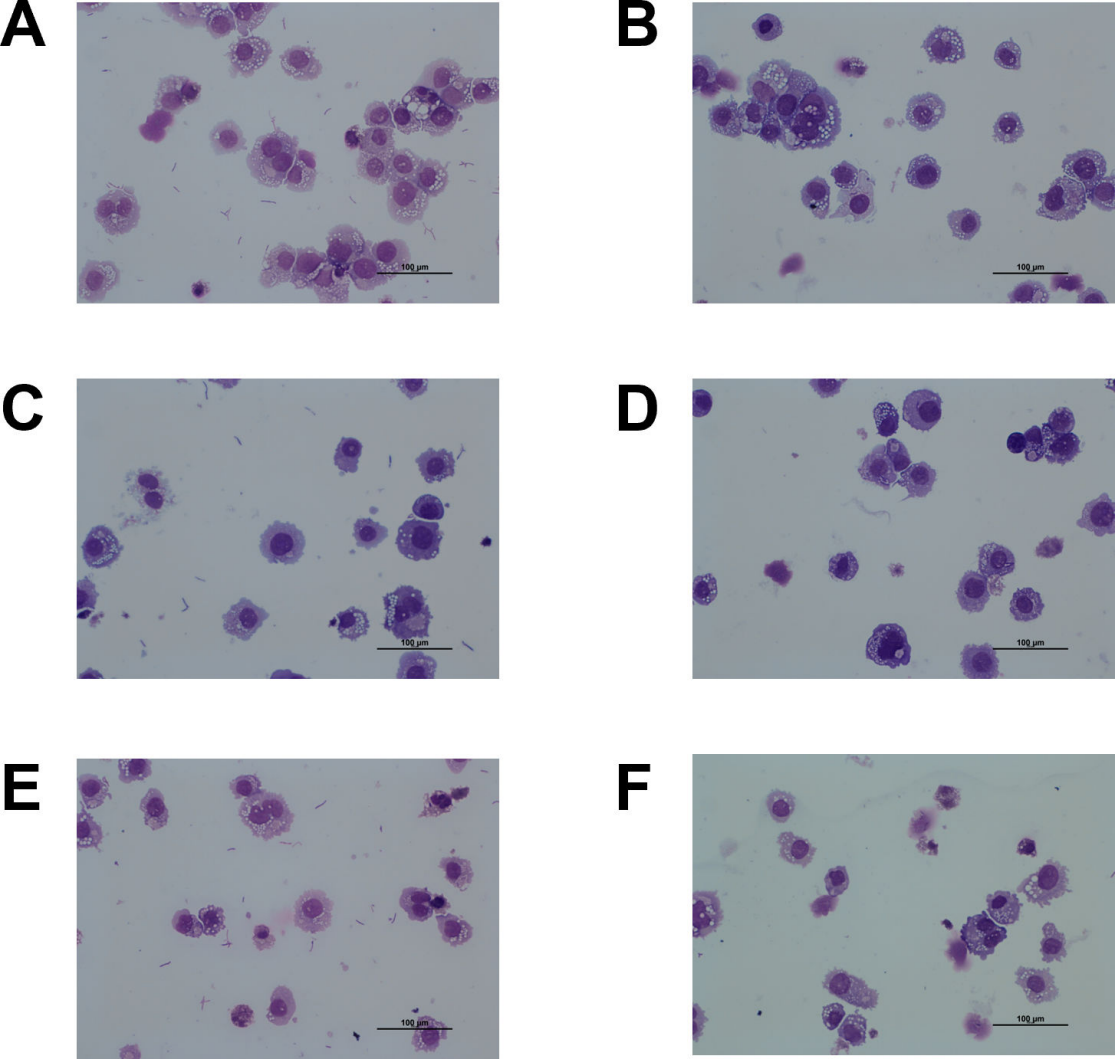
Microscopic examination of THP-1 macrophages (ATCC TIB-202) infected with *Rhodococcus equi* ATCC 33701 and exposed for 40 h to (**A and B**) doxycycline (10 µg/mL), (**C and D**) gamithromycin (5 µg/mL), and (**E and F**) doxycycline (10 µg/mL) + gamithromycin (5 µg/mL). Panels A, C, and E show the cells before the washing steps, and panels B, D, and F show the cells after the washing steps. Suspensions of cells were centrifuged on slides and stained with May-Grünwald-Giemsa stain.

We assessed the efficacy of the washing protocol based on microscopy for doxycycline and gamithromycin alone and their combination (Fig. 2). Before washing, bacteria were visible in the extracellular environment for both antibiotics and their combination (Fig. 2A, C and E). After three washes with PBS, we did not detect extracellular bacteria; however, some bacteria remained visible within the vacuoles of THP-1 cells exposed to doxycycline and gamithromycin alone or their combination (Fig. 2B, D and F). This method was thus subsequently applied to assess the intracellular activity of rifampicin, doxycycline, and macrolides, as a complement to the evaluation of their total activity (including both intracellular and extracellular components).

### Total (intracellular and extracellular) bactericidal activity of rifampicin, doxycycline, and macrolides alone or combined on *R. equi*

**Fig 3:**
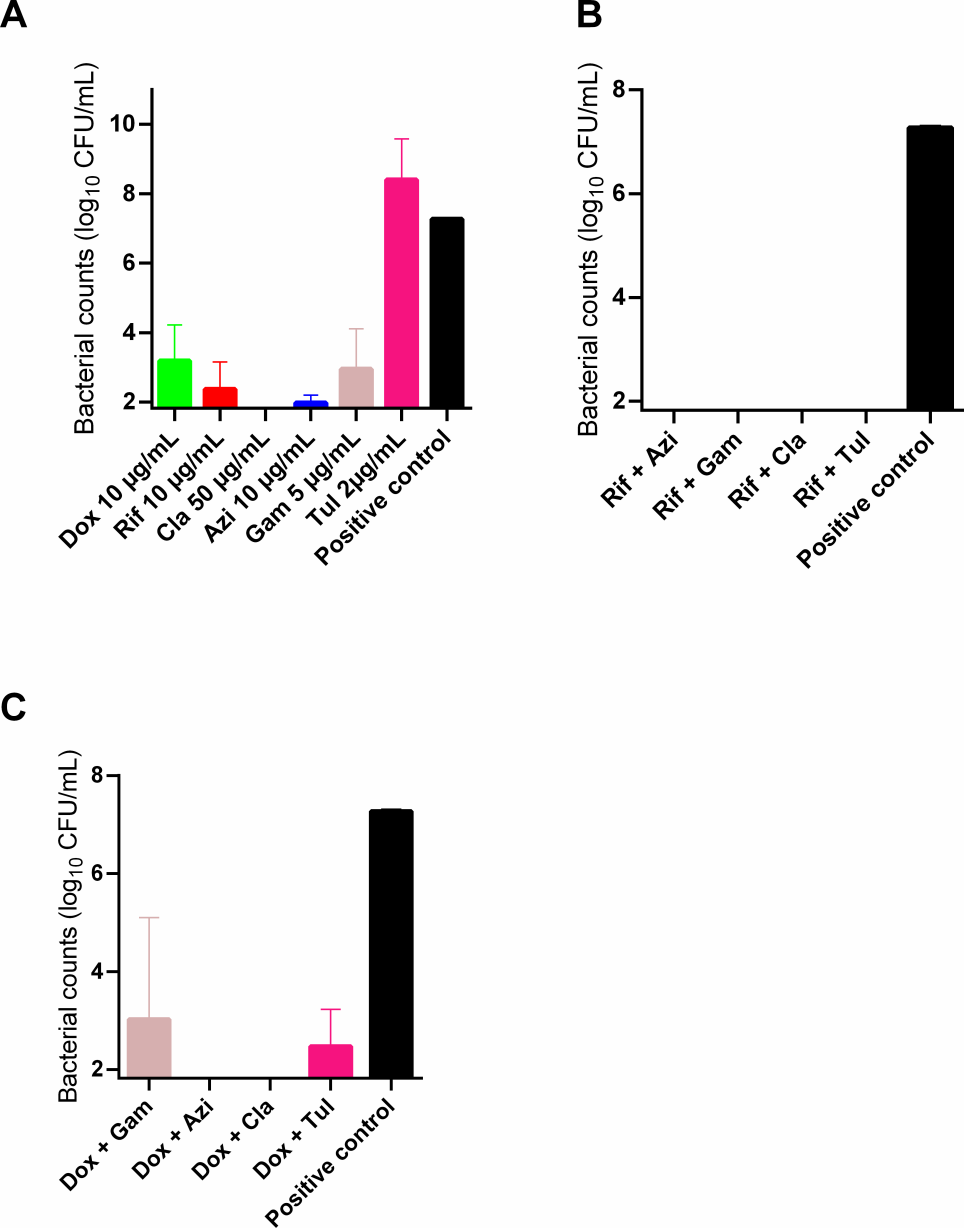
The total count of (extracellular and intracellular) *Rhodococcus equi* after phagocytosis by THP-1 macrophages (ATCC TIB-202) for 6 h, followed by exposure for 40 h to (**A**) doxycycline (Dox, 10 µg/mL), rifampicin (Rif, 10 µg/mL), clarithromycin (Cla, 50 µg/mL), azithromycin (Azi, 10 µg/mL), gamithromycin (Gam, 5 µg/mL), or tulathromycin (Tul, 2 µg/mL) alone; (**B**) rifampicin in combination with macrolides; and (**C**) doxycycline in combination with macrolides. Each value represents the mean of three independent experiments. All antibiotics alone, except tulathromycin, and all combinations are significantly different from the positive control (*P* < 0.05). The limit of quantification is 1.8 log_10_ CFU/mL.

In order to characterize the therapeutic potential of rifampicin, doxycycline, and macrolides against *R. equi* infections, we first evaluated their overall efficacy in the macrophage infection assay. Total bacterial counts, after a 6-h phagocytosis period, followed by an additional 40 h in the presence of antibiotics, either individually or in combination are represented in Fig. 3.

When tested alone, only clarithromycin completely eradicated both extracellular and intracellular *R. equi* at a concentration achievable in foals. Doxycycline, rifampicin, azithromycin, and gamithromycin significantly reduced the bacterial load, with a count ranging from 3 × 10^2^ to 1 × 10^2^ CFU/mL after exposure for 40 h. In contrast, tulathromycin showed minimal efficacy and no bacterial reduction, with a remaining bacterial count of around 1 × 10^8^ CFU/mL, close to the positive control (Fig. 3A). Macrolides combined with rifampicin demonstrated high efficacy against *R. equi*, reducing the total bacterial count to below the limit of quantification (Fig. 3B).

The combination of doxycycline with either clarithromycin or azithromycin reduced the bacterial count to below the limit of quantification. In contrast, combining doxycycline with gamithromycin did not achieve complete bacterial eradication, with the total bacterial count remaining at 1 × 10^3^ CFU/mL. Similarly, the combination of doxycycline with tulathromycin was less effective, leaving a residual bacterial count of 2.5 × 10^2^ CFU/mL (Fig. 3C).

### Intracellular bactericidal activity of rifampicin, doxycycline, and macrolides alone or combined on *R. equi*

**Fig 4:**
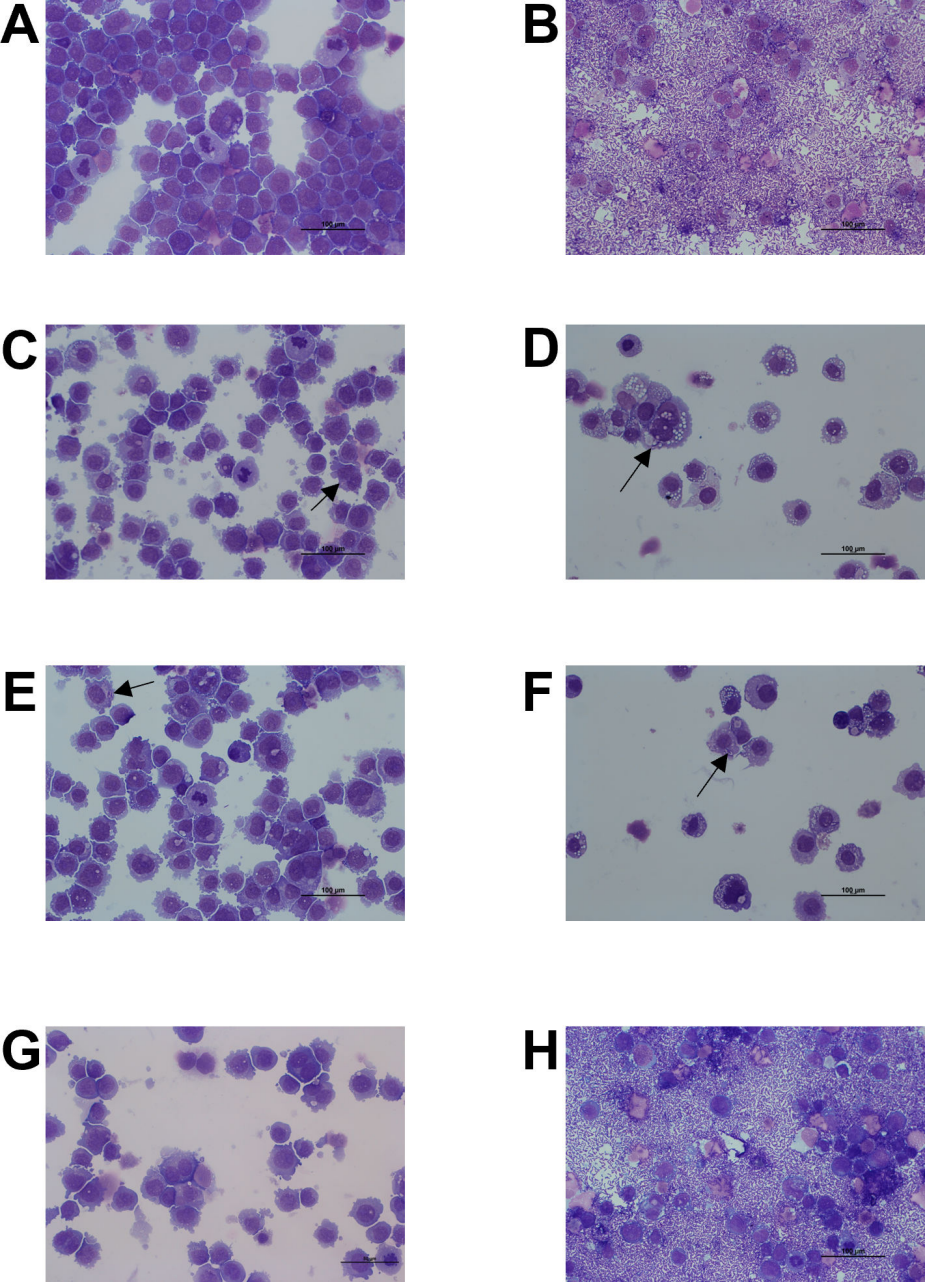
Microscopic examination of THP-1 macrophages infected with *Rhodococcus equi* ATCC 33701 and exposed to one antibiotic for 40 h followed by three washes. Suspensions of cells were centrifuged on slides and stained with May-Grünwald-Giemsa stain. (**A**) Negative control. (**B**) Positive control: phagocytosis for 46 h. Phagocytosis for 6 h followed by treatment for 40 h with (**C**) rifampicin (10 µg/mL), (**D**) doxycycline (10 µg/mL) (image reused from Fig. 1B), (**E**) azithromycin (10 µg/mL), (**F**) gamithromycin (5 µg/mL), (**G**) clarithromycin (50 µg/mL) (image reused from Fig. 1D), and (**H**) tulathromycin (2 µg/mL).

In addition to the determination of total bacteria, we also counted (Fig. S1) and observed intracellular bacteria by optical microscopy after the washing protocol. In microscopy images, only clarithromycin eradicated intracellular bacteria, with no bacteria detected in THP-1 cells, similarly to the negative control without bacteria (Fig. 4A and G). Doxycycline, rifampicin, azithromycin, and gamithromycin reduced the bacterial load, with only a few bacteria observed within cellular vacuoles (Fig. 4C through F). In contrast, tulathromycin demonstrated no efficacy, with many bacteria observed both inside and outside the cells, comparable to the positive control (Fig. 4B and H).

**Fig 5:**
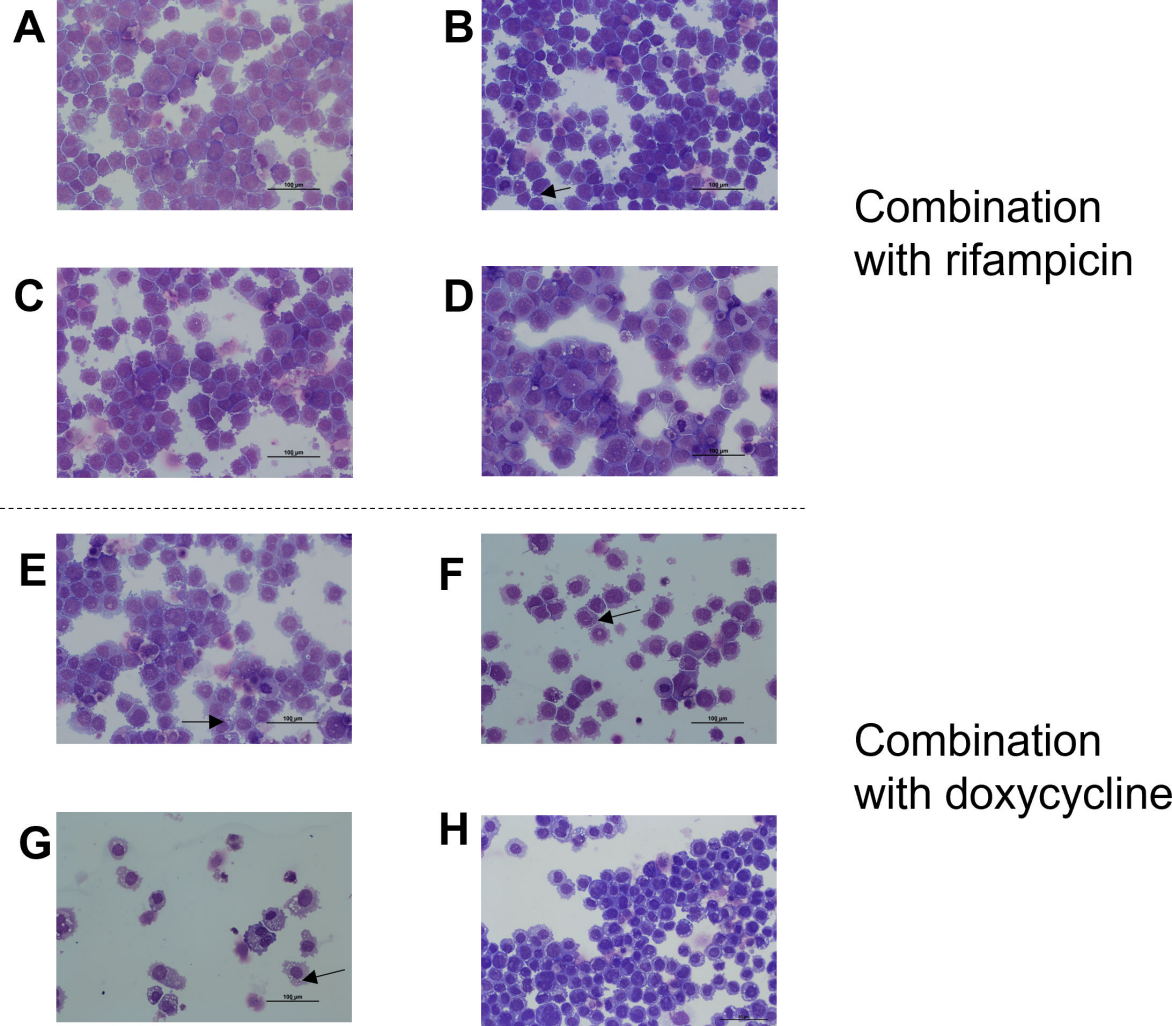
Microscopic examination of THP-1 macrophages infected with *Rhodococcus equi* ATCC 33701 and exposed to antibiotic combinations for 40 h followed by three washes. Suspensions of cells were centrifuged on slides and stained with May-Grünwald-Giemsa stain. Phagocytosis for 6 h followed by treatment for 40 h with (**A**) rifampicin (10 µg/mL) + azithromycin (10 µg/mL), (**B**) rifampicin (10 µg/mL) + tulathromycin (2 µg/mL), (**C**) rifampicin (10 µg/mL) + gamithromycin (5 µg/mL), (**D**) rifampicin (10 µg/mL) + clarithromycin (50 µg/mL), (**E**) doxycycline (10 µg/mL) + azithromycin (10 µg/mL), (**F**) doxycycline (10 µg/mL) + tulathromycin (2 µg/mL), (**G**) doxycycline (10 µg/mL) + gamithromycin (5 µg/mL) (image reused from Fig. 1F), and (**H**) doxycycline (10 µg/mL) + clarithromycin (50 µg/mL).

Combination of rifampicin and macrolides exhibited high efficacy against *R. equi*, with no bacteria visible in THP-1 cells (Fig. 5A, C and D). The only exception was the combination with tulathromycin, where a few bacteria were still visible in vacuoles (Fig. 5B). The combination of clarithromycin and doxycycline was highly effective, with no bacteria visible in THP-1 cells (Fig. 5H). However, the combination of doxycycline with azithromycin, gamithromycin, or tulathromycin was less effective, as we still detected a few bacteria in THP-1 cells (Fig. 5E through G).

## DISCUSSION

The primary objective of this study was to evaluate the activity of rifampicin, doxycycline, and four macrolides on both extracellular and intracellular *R. equi*. Unlike obligate intracellular bacteria, such as Chlamydiae, *R. equi* can replicate both intracellularly and extracellularly, and antibiotics must be effective in both environments. The relative ease of replication of the bacteria in each environment likely depends on specific conditions. Consequently, the ratio of extracellular to intracellular bacteria may differ significantly between *in vitro* and *in vivo* contexts, making it quite difficult to determine the exact proportions of extracellular and intracellular bacteria during *R. equi* infections in foals. However, this uncertainty does not undermine the relevance of *in vitro* experiments, provided that they demonstrate that the antibiotic is effective against both extracellular and intracellular bacteria.

We initially attempted to apply published methods to count intracellular *R. equi*, but we found that approaches validated for other bacterial species might not be suitable for *R. equi* and that the assessment of intracellular activity of antibiotics on *R. equi* proved to be challenging.

For gentamicin, vancomycin, and amikacin, the MICs obtained in this study are consistent with previously reported MICs in standard media (14, 28) and are 8–200-fold below the concentrations typically used for extracellular bacteria eradication. Time-kill studies were conducted over 24 h using antibiotic concentrations previously described in the literature to result in extracellular eradication (13, 15–18) without supporting data for *R. equi*. In our study, these concentrations showed a lack of efficacy in eradicating *R. equi* within 1 or even 8 h. Indeed, Giguère et al. (14) also demonstrated that gentamicin failed to completely eliminate extracellular *R. equi*, raising doubts about its reliability in experimental setups. Here, we confirmed a bacteriostatic activity of gentamicin against *R. equi* at 10 and 100 µg/mL for the first 8 h with a failure to eradicate *R. equi* even after 24 h. For vancomycin and amikacin, complete bacterial eradication was only achieved after 24 h of exposure. This highlights the ineffectiveness of bactericidal drugs in rapidly eliminating extracellular *R. equi* within shorter critical periods, such as 1 or 2 h. Given that the generation time of *R. equi* exceeds 1 h (19), the limited immediate efficacy of these antibiotics is not surprizing. In contrast, *E. coli* and *S. aureus* have much shorter generation times of approximately 20 min (29, 30), allowing three replication cycles within 1 h, which probably enables antibiotics to effectively target and eradicate them. The delayed action of vancomycin and amikacin observed in this study posed a significant issue in phagocytosis assays. During the 24-h timeframe required for these antibiotics to exert their antimicrobial action, *R. equi* residing intracellularly can replicate and cause host cell lysis, subsequently releasing bacteria into the extracellular environment and compromising the accuracy of experimental counts. Moreover, the longer the exposure time, the higher the risk of intracellular penetration of the drugs. Studies have demonstrated that after exposure for 24 h, the cellular-to-extracellular concentration ratio for gentamicin is approximately 6.3 in macrophages (31), and after several days the ratio is 2–4 (32). Thus, the reliance on these antibiotics to distinguish between intracellular and extracellular bacteria raises some issues, and the potential incomplete elimination of extracellular bacteria by amikacin may have led to an overestimation of intracellular bacterial counts (14).

Here, we proposed an alternative method to the recourse of bactericidal drugs to approximate extracellular and intracellular activity of antibiotics at concentrations achievable in the PELF of foals. To investigate intracellular antibiotic activity against *R. equi*, we used THP-1 human macrophage-like cells, a well-established and reproducible *in vitro* model for studying intracellular infections (33). These cells efficiently phagocytose *R. equi* (34), and thus provide a standardized cellular environment suitable for assessing antibiotic efficacy within host cells, assuming that antibiotic penetration and activity are comparable to those in foal macrophages. After phagocytosis and exposure to the tested antibiotics, we performed several washing steps, and it appears to remove the remaining extracellular bacteria detectable by microscopic examination before washes, enabling accurate counting of intracellular bacteria. We successfully implemented this method to compare antibiotics commonly used against *R. equi* infections, although it presented a minor limitation of the washing procedure for conditions, such as exposure to tulathromycin, with very high bacterial loads (exceeding 10^8^ CFU/mL) after drug exposure. Indeed, we were able to prove, based on bacterial counts and microscopy, that tulathromycin has very limited activity on extracellular bacteria, but our method cannot definitively conclude on the efficacy of tulathromycin on intracellular bacteria.

For other antibiotics with higher activity against *R. equi*, the bacterial counts obtained with our method were in accordance with those obtained in our previous study conducted on extracellular bacteria by mimicking intracellular or extracellular conditions (11). Among the tested antibiotics, clarithromycin alone demonstrated, based on bacterial counts and microscopy, the ability to eradicate extracellular and intracellular *R. equi*. Gamithromycin, azithromycin, doxycycline, and rifampicin reduced the total bacterial load to below 10^3^ CFU/mL after exposure for 40 h. Eradication of both extracellular and intracellular bacteria was also observed with combinations of azithromycin or gamithromycin with rifampicin. The effect of the combination of doxycycline with azithromycin and gamithromycin on both bacterial populations was only slightly lower than that observed for the combination with rifampicin, suggesting that doxycycline could potentially substitute rifampicin in the current standard treatments.

In a previous study (11), we demonstrated that the activity of macrolides against R. equi could be influenced by vacuolar pH. Although the exact pH of the *R. equi*–containing vacuoles remains unclear (5), our findings confirm intracellular penetration and activity of macrolides, with the exception of tulathromycin, as evidenced by the reduction of intracellular bacteria.

In conclusion, this study emphasizes the need for methods specifically developed to assess antibiotic efficacy against *R. equi*. Here, we proposed washing steps to eliminate extracellular bacteria; however, methods involving bactericidal drugs could also be further refined and validated to specifically suit *R. equi*. Additionally, alternative approaches, such as the use of fluorescently labeled *R. equi* strains combined with advanced imaging techniques, could be employed to directly quantify intracellular bacterial load. This will then enable the proposal of effective antibiotics against *R. equi* infections based on reliable *in vitro* experiments. By refining our understanding of antibiotic activity against intracellular *R. equi*, this will ultimately limit the need for more challenging clinical trials and improve the overall health and well-being of affected animals.

## SUPPLEMENTAL MATERIAL

Fig. S1 spectrum.01205-25-s0001.pdf Counts of intracellular *Rhodococcus equi* ATCC 33701 after phagocytosis by THP-1 3 macrophages for 6 h, followed by exposure for 40 h and three washes.

ASM does not own the copyrights to Supplemental Material that may be linked to, or accessed through, an article. The authors have granted ASM a non-exclusive, world-wide license to publish the Supplemental Material files. Please contact the corresponding author directly for reuse.

## References

[1] Barton MD, Hughes KL. 1984. Ecology of Rhodococcus equi. Vet Microbiol 9:65–76. doi:10.1016/0378-1135(84)90079-8 6719819

[2] Cohen ND. 2014. Rhodococcus equi foal pneumonia. Vet Clin North Am Equine Pract 30:609–622. doi:10.1016/j.cveq.2014.08.010 25282322

[3] Prescott JF. 1991. Rhodococcus equi: an animal and human pathogen. Clin Microbiol Rev 4:20–34. doi:10.1128/CMR.4.1.20 2004346 PMC358176

[4] Reuss SM, Chaffin MK, Cohen ND. 2009. Extrapulmonary disorders associated with Rhodococcus equi infection in foals: 150 cases (1987-2007). J Am Vet Med Assoc 235:855–863. doi:10.2460/javma.235.7.855 19793018

[5] Anand T, Bera BC, Vaid RK, Shanmugasundaram K, Sharma G, Virmani N, Shukla BN, Barua S, Malik P, Singh RK. 2014. Molecular characterization of virulence-associated protein (Vap) family genes of pathogenic Rhodococcus equi isolates from clinical cases of Indian equines. Indian J Biotechnol 13

[6] Letek M, González P, Macarthur I, Rodríguez H, Freeman TC, Valero-Rello A, Blanco M, Buckley T, Cherevach I, Fahey R, Hapeshi A, Holdstock J, Leadon D, Navas J, Ocampo A, Quail MA, Sanders M, Scortti MM, Prescott JF, Fogarty U, Meijer WG, Parkhill J, Bentley SD, Vázquez-Boland JA. 2010. The genome of a pathogenic Rhodococcus: cooptive virulence underpinned by key gene acquisitions. PLoS Genet 6:e1001145. doi:10.1371/journal.pgen.1001145 20941392 PMC2947987

[7] Takai S, Sekizaki T, Ozawa T, Sugawara T, Watanabe Y, Tsubaki S. 1991. Association between a large plasmid and 15- to 17-kilodalton antigens in virulent Rhodococcus equi. Infect Immun 59:4056–4060. doi:10.1128/iai.59.11.4056-4060.1991 1937765 PMC258996

[8] Sivaloganathan DM, Brynildsen MP. 2021. Phagosome-bacteria interactions from the bottom up. Annu Rev Chem Biomol Eng 12:309–331. doi:10.1146/annurev-chembioeng-090920-015024 33781082

[9] Giguère S, Lee EA, Guldbech KM, Berghaus LJ. 2012. In vitro synergy, pharmacodynamics, and postantibiotic effect of 11 antimicrobial agents against Rhodococcus equi. Vet Microbiol 160:207–213. doi:10.1016/j.vetmic.2012.05.031 22704561

[10] Carlson KL, Kuskie KR, Chaffin KM, Libal MC, Giguère S, Lawhon SD, Cohen ND. 2010. Antimicrobial activity of tulathromycin and 14 other antimicrobials against virulent Rhodococcus equi in vitro. Vet Ther 11:E1–9. 20957614

[11] Huguet A-S, Gourbeyre O, Bernand A, Philibert C, Bousquet-Melou A, Lallemand EA, Ferran AA. 2024. Comparative bactericidal activity of four macrolides alone and combined with rifampicin or doxycycline against Rhodococcus equi at concentrations achievable in foals. Front Pharmacol 15:1458496. doi:10.3389/fphar.2024.1458496 39624843 PMC11608945

[12] Bargen K, Scraba M, Krämer I, Ketterer M, Nehls C, Krokowski S, Repnik U, Wittlich M, Maaser A, Zapka P, Bunge M, Schlesinger M, Huth G, Klees A, Hansen P, Jeschke A, Bendas G, Utermöhlen O, Griffiths G, Gutsmann T, Wohlmann J, Haas A. 2019. Virulence‐associated protein A from Rhodococcus equi is an intercompartmental pH‐neutralising virulence factor. Cell Microbiol 21:e12958. doi:10.1111/cmi.12958 30251327

[13] von Bargen K, Polidori M, Becken U, Huth G, Prescott JF, Haas A. 2009. Rhodococcus equi virulence-associated protein A is required for diversion of phagosome biogenesis but not for cytotoxicity. Infect Immun 77:5676–5681. doi:10.1128/IAI.00856-09 19797071 PMC2786453

[14] Giguère S, Berghaus LJ, Lee EA. 2015. Activity of 10 antimicrobial agents against intracellular Rhodococcus equi. Vet Microbiol 178:275–278. doi:10.1016/j.vetmic.2015.05.019 26051479

[15] Mourenza Á, Gil JA, Mateos LM, Letek M. 2020. A novel screening strategy reveals ROS-generating antimicrobials that act synergistically against the intracellular veterinary pathogen Rhodococcus equi. Antioxidants (Basel) 9:114. doi:10.3390/antiox9020114 32012850 PMC7070597

[16] Willingham-Lane JM, Berghaus LJ, Berghaus RD, Hart KA, Giguère S. 2019. Effect of macrolide and rifampin resistance on fitness of Rhodococcus equi during intramacrophage replication and in vivo Infect Immun 87:e00281-19. doi:10.1128/IAI.00281-19 31331959 PMC6759311

[17] Hondalus MK, Mosser DM. 1994. Survival and replication of Rhodococcus equi in macrophages. Infect Immun 62:4167–4175. doi:10.1128/iai.62.10.4167-4175.1994 7927672 PMC303092

[18] Berghaus LJ, Giguère S, Sturgill TL. 2014. Effects of age and macrophage lineage on intracellular survival and cytokine induction after infection with Rhodococcus equi. Vet Immunol Immunopathol 160:41–50. doi:10.1016/j.vetimm.2014.03.010 24736188

[19] Bujold AR, Lani NR, Sanz MG. 2019. Strain-to-strain variation of Rhodococcus equi growth and biofilm formation in vitro. BMC Res Notes 12:519. doi:10.1186/s13104-019-4560-1 31426832 PMC6701102

[20] Halawa EM, Fadel M, Al-Rabia MW, Behairy A, Nouh NA, Abdo M, Olga R, Fericean L, Atwa AM, El-Nablaway M, Abdeen A. 2023. Antibiotic action and resistance: updated review of mechanisms, spread, influencing factors, and alternative approaches for combating resistance. Front Pharmacol 14:1305294. doi:10.3389/fphar.2023.1305294 38283841 PMC10820715

[21] M100-S11, Performance standards for antimicrobial susceptibility testing. 2001. Clin Microbiol Newsl 23:49. doi:10.1016/S0196-4399(01)88009-0

[22] Berlin S, Kirschbaum A, Spieckermann L, Oswald S, Keiser M, Grube M, Venner M, Siegmund W. 2017. Pharmacological indices and pulmonary distribution of rifampicin after repeated oral administration in healthy foals. Equine Vet J 49:618–623. doi:10.1111/evj.12662 28063154

[23] Womble A, Giguère S, Lee EA. 2007. Pharmacokinetics of oral doxycycline and concentrations in body fluids and bronchoalveolar cells of foals. Vet Pharm Therapeutics 30:187–193. doi:10.1111/j.1365-2885.2007.00857.x 17472649

[24] Giguère S. 2017. Treatment of infections caused by Rhodococcus equi. Vet Clin North Am Equine Pract 33:67–85. doi:10.1016/j.cveq.2016.11.002 28161038

[25] Jacks S, Gigue`re S, Gronwall RR, Brown MP, Merritt KA. 2002. Disposition of oral clarithromycin in foals. Vet Pharm Therapeutics 25:359–362. doi:10.1046/j.1365-2885.2002.00420.x 12423226

[26] Berghaus LJ, Giguère S, Sturgill TL, Bade D, Malinski TJ, Huang R. 2012. Plasma pharmacokinetics, pulmonary distribution, and in vitro activity of gamithromycin in foals. Vet Pharm Therapeutics 35:59–66. doi:10.1111/j.1365-2885.2011.01292.x 21443748

[27] Venner M, Peters J, Höhensteiger N, Schock B, Bornhorst A, Grube M, Adam U, Scheuch E, Weitschies W, Rosskopf D, Kroemer HK, Siegmund W. 2010. Concentration of the macrolide antibiotic tulathromycin in broncho-alveolar cells is influenced by comedication of rifampicin in foals. Naunyn Schmiedebergs Arch Pharmacol 381:161–169. doi:10.1007/s00210-009-0481-1 20012942

[28] Berghaus LJ, Giguère S, Guldbech K. 2013. Mutant prevention concentration and mutant selection window for 10 antimicrobial agents against Rhodococcus equi. Vet Microbiol 166:670–675. doi:10.1016/j.vetmic.2013.07.006 23915992

[29] Missiakas DM, Schneewind O. 2013. Growth and laboratory maintenance of Staphylococcus aureus. CP Microbiology 28. doi:10.1002/9780471729259.mc09c01s28 PMC6211185 23408134

[30] Tuttle AR, Trahan ND, Son MS. 2021. Growth and maintenance of Escherichia coli laboratory strains. Curr Protoc 1:e20. doi:10.1002/cpz1.20 33484484 PMC8006063

[31] Seral C, Van Bambeke F, Tulkens PM. 2003. Quantitative analysis of gentamicin, azithromycin, telithromycin, ciprofloxacin, moxifloxacin, and oritavancin (LY333328) activities against intracellular Staphylococcus aureus in mouse J774 macrophages. Antimicrob Agents Chemother 47:2283–2292. doi:10.1128/AAC.47.7.2283-2292.2003 12821480 PMC161849

[32] Bambeke FV, Barcia-Macay M, Lemaire S. 2006. Cellular pharmacodynamics and pharmacokinetics of antibiotics: current views and perspectives. Curr Opin Drug Discov Devel 9 16566292

[33] Carryn S, Van Bambeke F, Mingeot-Leclercq M-P, Tulkens PM. 2002. Comparative intracellular (THP-1 macrophage) and extracellular activities of beta-lactams, azithromycin, gentamicin, and fluoroquinolones against Listeria monocytogenes at clinically relevant concentrations. Antimicrob Agents Chemother 46:2095–2103. doi:10.1128/AAC.46.7.2095-2103.2002 12069960 PMC127291

[34] Rampacci E, Marenzoni ML, Chiaradia E, Passamonti F, Ricci M, Pepe M, Coletti M, Giovagnoli S. 2018. In vitro performances of novel co-spray-dried azithromycin/rifampicin microparticles for Rhodococcus equi disease treatment. Sci Rep 8:12149. doi:10.1038/s41598-018-30715-z 30108265 PMC6092326

