# Supplementary material for: Reassessment of extracellular and intracellular activity of macrolides, rifampicin, and doxycycline against Rhodococcus equi based on bacterial counts and microscopy
Source: Microbiol Spectr. 2025 Jul 29;13(9):e01205-25. doi: 10.1128/spectrum.01205-25 (PMC12403593; doi:10.1128/spectrum.01205-25)
Supplement: Fig. S1 — Counts of intracellular Rhodococcus equi ATCC 33701 after phagocytosis by THP-1 3 macrophages for 6 h, followed by exposure for 40 h and three washes. [file spectrum.01205-25-s0001.pdf]

Supplementary Data

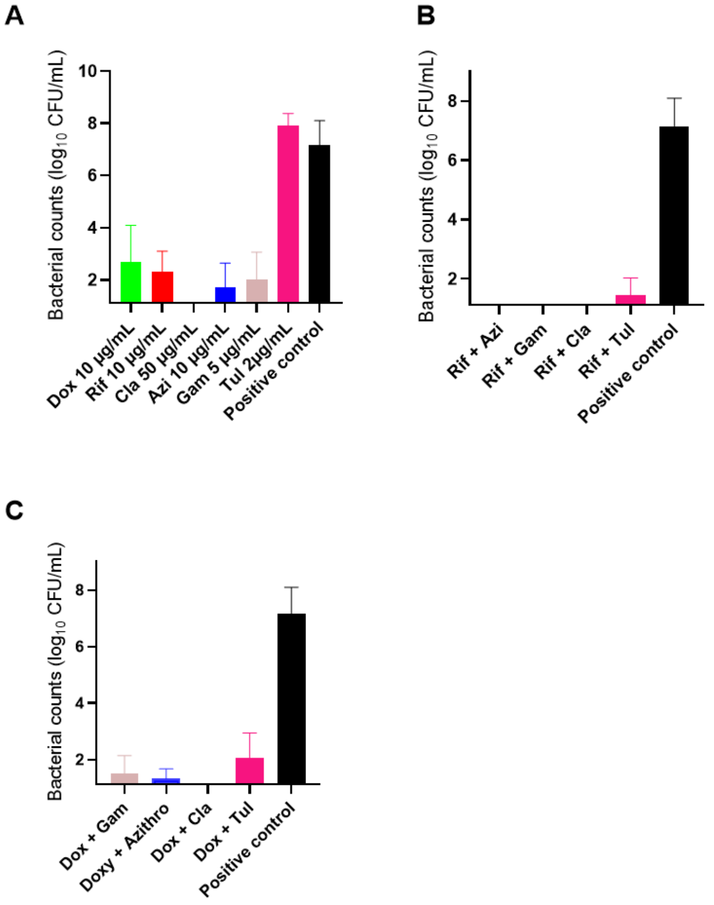

Figure S1: Counts of intracellular *Rhodococcus equi* ATCC 33701 after phagocytosis by THP-1 macrophages for 6 hours, followed by exposure for 40 hours and three washes. The tested antibiotics were (A) doxycycline (Dox, 10 µg/mL), rifampicin (Rif, 10 µg/mL), clarithromycin (Cla, 50 µg/mL), azithromycin (Azi, 10 µg/mL), gamithromycin (Gam, 5 µg/mL) or tulathromycin (Tul, 2 µg/mL) alone; (B) rifampicin in combination with macrolides; and (C) doxycycline in combination with macrolides. Each value represents the mean of three independent experiments. All antibiotics, except tulathromycin, and all combinations are significantly different from the positive control ($p < 0.05$). The limit of quantification is 1.1 log_{10} CFU/mL.
